# Peculiarities of the Super-Folder GFP Folding in a Crowded Milieu

**DOI:** 10.3390/ijms17111805

**Published:** 2016-10-28

**Authors:** Olesya V. Stepanenko, Olga V. Stepanenko, Irina M. Kuznetsova, Vladimir N. Uversky, Konstantin K. Turoverov

**Affiliations:** 1 Laboratory of Structural Dynamics, Stability and Folding of Proteins, Institute of Cytology, Russian Academy of Sciences, 4 Tikhoretsky Ave., St. Petersburg 194064, Russia; lvs@incras.ru (Ole.V.S.); sov@incras.ru (Olg.V.S.); imk@incras.ru (I.M.K.); 2 Institute of Physics, Nanotechnology and Telecommunications, Peter the Great St. Petersburg State Polytechnic University, 29 Polytechnicheskaya st., St. Petersburg 195251, Russia; 3 Department of Molecular Medicine and USF Health Byrd Alzheimer’s Research Institute, Morsani College of Medicine, University of South Florida, 12901 Bruce B. Downs Blvd. MDC07, Tampa, FL 33612, USA

**Keywords:** super-folder GFP, protein folding, conformational stability, crowded milieu, excluded volume effect, solvent properties

## Abstract

The natural cellular milieu is crowded by large quantities of various biological macromolecules. This complex environment is characterized by a limited amount of unoccupied space, limited amounts of free water, and changed solvent properties. Obviously, such a tightly packed cellular environment is poorly mimicked by traditional physiological conditions, where low concentrations of a protein of interest are analyzed in slightly salted aqueous solutions. An alternative is given by the use of a model crowded milieu, where a protein of interest is immersed in a solution containing high concentrations of various polymers that serve as model crowding agents. An expected outcome of the presence of such macromolecular crowding agents is their ability to increase conformational stability of a globular protein due to the excluded volume effects. In line with this hypothesis, the behavior of a query protein should be affected by the hydrodynamic size and concentration of an inert crowder (i.e., an agent that does not interact with the protein), whereas the chemical nature of a macromolecular crowder should not play a role in its ability to modulate conformational properties. In this study, the effects of different crowding agents (polyethylene glycols (PEGs) of various molecular masses (PEG-600, PEG-8000, and PEG-12000), Dextran-70, and Ficoll-70) on the spectral properties and unfolding–refolding processes of the super-folder green fluorescent protein (sfGFP) were investigated. sfGFP is differently affected by different crowders, suggesting that, in addition to the expected excluded volume effects, there are some changes in the solvent properties.

## 1. Introduction

Fluorescent protein markers (FPs) have become a valuable tool for solving many problems of molecular and cell biology using fluorescence microscopy [1,2,3,4,5,6,7]. The green fluorescent protein (GFP)-like fluorescent proteins have a special place among them, as they are relatively small proteins with a β-barrel structure containing a unique chromophore that absorbs and emits in the visible spectral region [8]. Compared to the well-known enhanced green fluorescent protein (EGFP), the amino acid sequence of a super-folder GFP (sfGFP) has nine amino acid substitutions. This protein does not have the drawbacks of other FPs, such as the tendency to aggregate and the irreversibility of their denaturation [9,10,11,12]. sfGFP is characterized by high efficiency of its folding, as it is able to fold even being fused to a poorly-folded partner peptide [10]. Furthermore, sfGFP in such a fusion construct increases the folding efficiency of its partner that it can be used for the expression of poorly-soluble recombinant proteins and peptides [13,14]. These properties of sfGFP defined the use of this protein as a convenient model subject for the folding-unfolding study of FPs.

We have previously investigated the equilibrium unfolding-folding processes of sfGFP induced by guanidine thiocyanate (GTC) in vitro [15,16]. The GTC-induced unfolding of sfGFP was shown to be reversible. This was confirmed by the complete recovery of all characteristics recorded for the pre-denatured sfGFP after refolding of this protein from its completely unfolded state. During the protein refolding, an off-pathway intermediate state was shown to be formed in the presence of the moderate denaturant concentrations. In this intermediate state, the β-barrel structure was almost completely formed, but the inner α-helix bearing the chromophore and a single tryptophan residue of the protein (Trp57) were not properly incorporated into the protein matrix. As for other FPs analyzed so far, the unfolding-refolding processes of sfGFP was characterized by hysteresis. The hysteretic behavior of FPs at their unfolding-refolding process is determined by the presence of the chromophore [12,16,17,18,19]. Indeed, folding of sfGFP in native conditions (after the protein biosynthesis) occurs in the absence of any chromophore (which happens at the latter maturation stage), whereas the protein undergoing refolding from the fully unfolded state already bears the mature chromophore (which, being a covalent adduct, is not destroyed by high concentrations of strong denaturants). Ones being formed, the chromophore strongly contributes not only to the protein refolding processes, but also to the protein conformational stability, by holding a compact structure of the β-barrel through non-covalent interactions with the surrounding protein matrix [15]. Notably, while studying the structural perturbations of FPs, and sfGFP in particular, the chromophore serves as a convenient and reliable marker of protein structural integrity, since it has a high quantum yield, only being properly incorporated into the β-barrel [3,16,20,21,22].

An interesting feature of FPs is a specific change in the spectral properties of the protein chromophore in the presence of ionic denaturants and salts [15,23], which should be considered in the study of the folding of these proteins. In fact, these constituents (ionic denaturants and salts) expressed strong influence on the spectral characteristics of the chromophore of sfGFP, without inducing disruption of the integrity of the protein structure [15,23]. The binding of an anion of the salt or ionic denaturant near the chromophore shifts the equilibrium between the neutral and the anionic forms of the chromophore and is not associated with the appearance of any partially-folded intermediates during the protein unfolding.

Since FPs are widely used as markers of various cellular processes, the study of the structural and spectral properties of these proteins under conditions simulating those found in the cellular environment; i.e., under macromolecular crowding, is of great interest. This crowded environment is characterized by the limited space available for a protein [24,25] and by the restricted amount of free water [24,26]. These environmental perturbations significantly influence a variety of biologically-relevant processes, such as the folding process, the binding of small molecules, enzymatic activity, protein-protein interactions, amyloid formation, etc. [24,27,28,29,30,31,32]. In laboratory practice, different polymer molecules, such as polyethylene glycol (PEG) of different molecular weights, Dextran, Ficoll, and inert proteins, are typically used as crowding agents [25,28,29,31]. We, and others, established that the excluded volume effect is not the only factor influencing the behavior of the protein in a crowded milieu [29,33,34]. Apparently, the special role belongs to the ability of the crowding agents to alter the properties of the solvent. This conclusion is based on the analysis of the urea-induced unfolding behavior of globular proteins belonging to different structural classes and having different physicochemical properties in the presence of the two commonly used crowding agents, PEG-8000 and Dextran-70. Particularly, our studies showed that globular proteins can be divided into the three categories: highly-stable proteins with no influence of the crowding agents, proteins markedly stabilized by polymers, and proteins appreciably destabilized by at least one of the crowding agents. Therefore, different polymers carry very different effects on the conformational stability of a protein. In the study of the folding-unfolding processes of bovine odorant-binding protein in the crowded conditions it was found out that the effect of the crowding agent PEG on the native protein structure, and its stability, strictly depends on the molecular weight of this crowder [35].

In the present study, we investigated the effect of different crowding agents on the spectral properties and unfolding-refolding processes of sfGFP induced by the GTC. PEGs of various molecular masses (PEG-600, PEG-8000, and PEG-12000), Dextran-70, and Ficoll-70, were used as crowding agents in this study.

## 2. Results

### 2.1. Spectral Properties of sfGFP in the Presence of Crowding Agents

The impact of crowding agents on the structure and spectral properties of sfGFP was studied first. To this end, we recorded the absorption spectra in the UV and visible region (i.e., the absorption originating from the tryptophan residue and the chromophore of the protein), the tryptophan fluorescence spectra and the fluorescence of the green chromophore (at λ_ex_ = 390 nm), the fluorescence excitation spectra of the chromophore (at λ_reg_ = 540 nm), the CD spectra in the far- and near-UV and visible regions. PEGs of different molecular weights (600, 8000, and 12,000 Da), Dextran-70, and Ficoll-70 were used as crowding agents. The action of crowding agents in low, medium, and high concentrations was tested.

The absorbance of protein samples did not exceed 0.15 in all of our experiments unless otherwise indicated. Taking into account the extinction coefficient ɛ_280_ = 31,519 M^−1^·sm^−1^ [19], this means that protein concentration was not higher than 5 µM. Therefore, we assumed that sfGFP existed as a monomer at our experimental conditions (low protein concentrations were needed for the spectroscopic characterization). It is worth noting that the monomerization of avGFP requires the disruption of the hydrophobic surface of the protein at the dimeric interface by introducing a set of charged residues in the key positions 206, 221, and 223. The sfGFP has hydrophobic residues Val 206, Leu 221, and Phe 223 at these positions (PDB file 2B3P [10]). This may be a reason for the sfGFP dimerization, especially at the conditions that favor aggregation, such as macromolecular crowding conditions. However, at the protein concentrations used in our studies, the aforementioned hydrophobic surface of sfGFP does not prevent a complete protein refolding in the absence of crowding agents [15]. Furthermore, it was shown that sfGFP behaves as a monomer in the cell [36].

**Figure 1:**
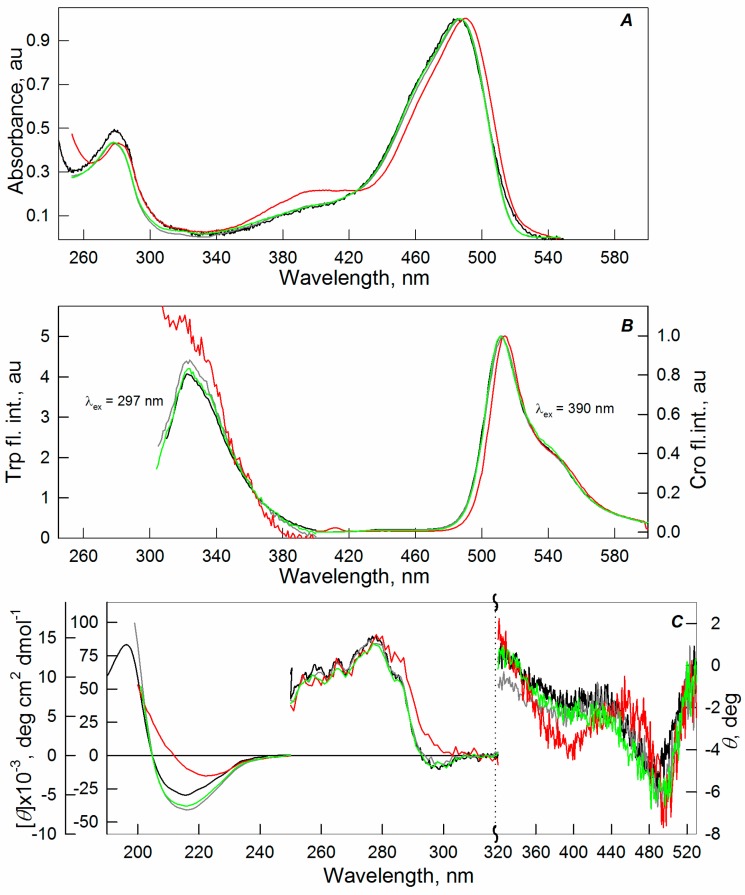
The effect of crowding agent PEG-12000 on the spectral properties of sfGFP. Absorption spectra (**A**); tryptophan fluorescence spectra at λ_ex_ = 295 nm and the fluorescence spectra of the protein chromophore at λ_ex_ = 390 nm (**B**); and CD spectra in the far (the data are represented in units of the molar ellipticity per amino acid residue), near (in units of the molar ellipticity), and visible UV region (in units of the ellipticity) were recorded (**C**). The applied concentrations of PEG-12000 were 8% (green line), 20% (gray line), and 30% (red line). The spectra of sfGFP in the buffered solution (black lines) are shown for comparison.

We found no significant changes in the spectral characteristics of sfGFP in the presence of Dextran-70 (see Supplementary Materials, Figure S1) and Ficoll-70 (see Supplementary Materials, Figure S2), PEG-600 (Figure S3), and PEG-8000 (Figure S4) at all tested concentrations of the crowding agents and in the presence of PEG-12000 at concentrations below 20% (Figure 1). Some reduction in the intensity of the UV-absorption band of sfGFP can be observed in the presence of Ficoll-70 (Figure S2). This is probably due to the fact that the crowding agent absorbs strongly in this spectral region (data not shown). It should be noted that the absorption spectra of sfGFP in the presence of 30% of PEG-8000 and of 20%–30% of PEG-12000 demonstrate a marked increase in light scattering. The strong increase in light scattering for the protein solution in the presence of 30% of PEG-12000 led to a significant distortion of the absorption spectrum of the protein. The protein solutions at these conditions were opalescent.

In addition to the increase in light scattering, an evident change in the spectral properties of sfGFP in the presence of 30% of PEG-12000 can be seen (Figure 1). At these conditions, there was some observed increase in the intensity of the shoulder at 390 nm and a slight redshift of the peak at 490 nm in the absorption, excitation, and CD spectra in the visible region of sfGFP. The far-UV CD spectra and the tryptophan fluorescence spectra of sfGFP were significantly distorted in the presence of high concentrations of PEG-12000. The decrease in the intensity of absorption and fluorescence of the tryptophan residue and the chromophore of sfGFP can also be observed. These effects become even more pronounced with the two-fold increase in the protein concentration (data not shown). Therefore, the aforementioned spectral changes of sfGFP under these conditions can be attributed to the aggregation of protein molecules.

The protein aggregation probably leads to shielding and scattering of the light beam, which resulted in a decrease in the proportion of light transmitted through the protein solution. This may decrease the absorption and fluorescence of a sample. As it is known, the light scattering can lead to a reduced CD signal. In our case, the strong light scattering resulted in the distortion of the CD signal of sfGFP. The deformation of sfGFP β-barrel induced by its aggregation can affect the network of intramolecular hydrogen bonds between the chromophore and the side chains of the residues that form the β-barrel of sfGFP. This, in turn, may shift the equilibrium between protein molecules containing neutral and anionic forms of the chromophore [23], resulting in an increase of the peak at 390 nm in the absorption, excitation, and the visible CD spectra of sfGFP. The redshift of the peak at 490 nm in the absorption spectra of sfGFP testifies for the stabilizing of the anionic chromophore of the protein [23,37,38].

### 2.2. Unfolding-Refolding of sfGFP in the Crowded Milieu

**Figure 2:**
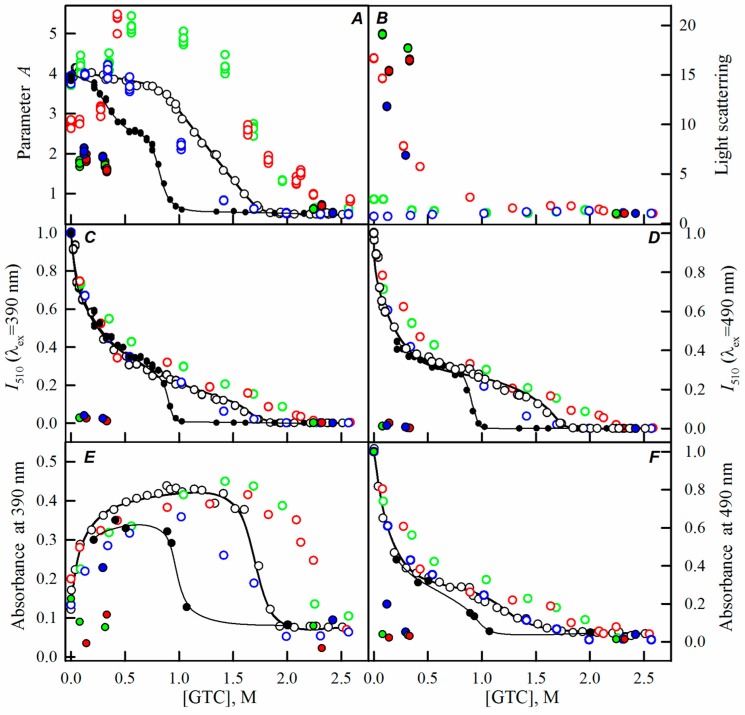
The effect of crowding agent PEG-12000 on the sfGFP conformational changes induced by GTC. Changes of parameter *A* (**A**); light scattering (**B**); corrected fluorescence intensity of green chromophore at two wavelengths of excitation of 390 nm (**C**) and 490 nm (**D**); and changes of absorbance at 390 nm (**E**) and 490 nm (**F**) are shown. Open symbols indicate unfolding and filled symbols represent refolding. Measurements were performed after 24 h incubation of native or denatured protein in the presence of GTC or GTC and the crowding agent. The applied concentrations of PEG-12000 were 8% (blue circles), 20% (green circles), and 30% (red circles). The unfolding-refolding curves of sfGFP in the absence of the crowding agent are also shown (black circles and lines).

While studying the processes of sfGFP folding-unfolding induced by GTC in the absence of crowding agents, we have shown that the disruption of the protein structure occurs within the 0.9–1.7 M GTC range (Figure 2, black lines and symbols). Observed changes of all recorded parameters of sfGFP at a GTC concentration below 0.7 M are caused by the binding of a negatively-charged SCN^−^ ions in the vicinity of the chromophore with subsequent redistribution of the protein molecules bearing the chromophore in the anionic and neutral forms, the inhibition of proton transfer in the excited state of the chromophore, and the quenching of the chromophore fluorescence by the sulfur atom of SCN^−^ ions [15,23].

**Figure 3:**
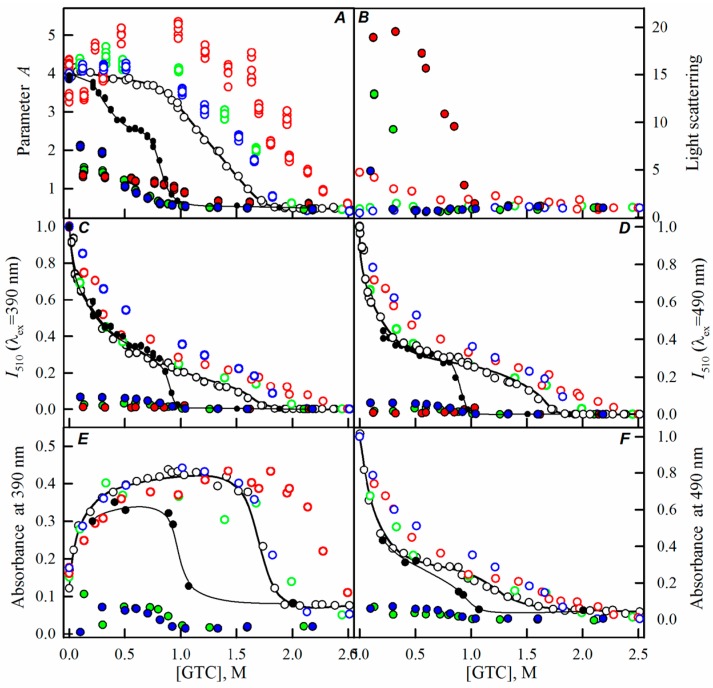
The effect of crowding agent PEG-8000 on the sfGFP conformational changes induced by GTC. For more details see the legend of Figure 2. Applied concentrations of PEG-8000 were 8% (blue circles), 12% (green circles), and 30% (red circles).

**Figure 4:**
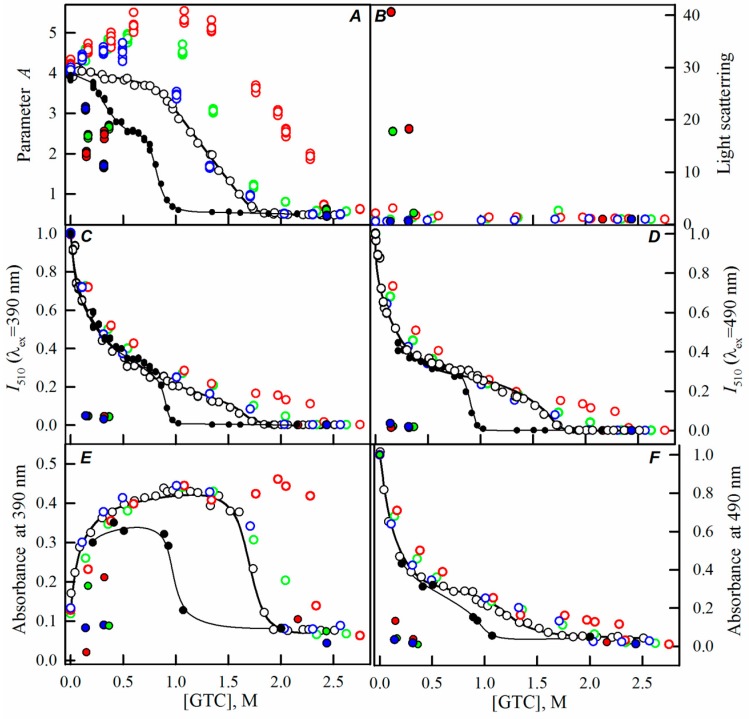
The effect of crowding agent PEG-600 on the sfGFP conformational changes induced by GTC. For more details see the legend of Figure 2. Applied concentrations of PEG-600 were 8% (blue circles), 20% (green circles), and 30% (red circles).

**Figure 5:**
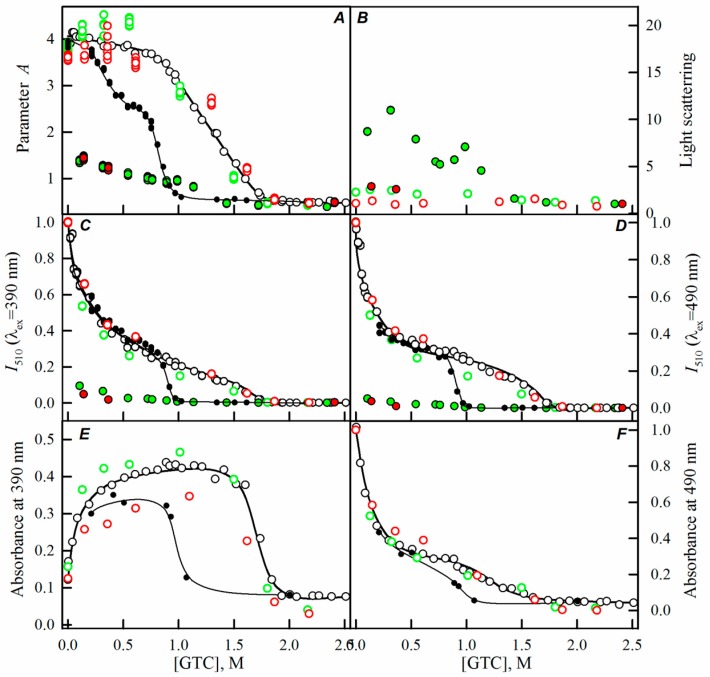
The effect of crowding agent Dextran-70 on the conformational changes of sfGFP induced by GTC. For more details see the legend of Figure 2. Applied concentrations of Dextran-70 were 24% (green circles), and 30% (red circles).

**Figure 6:**
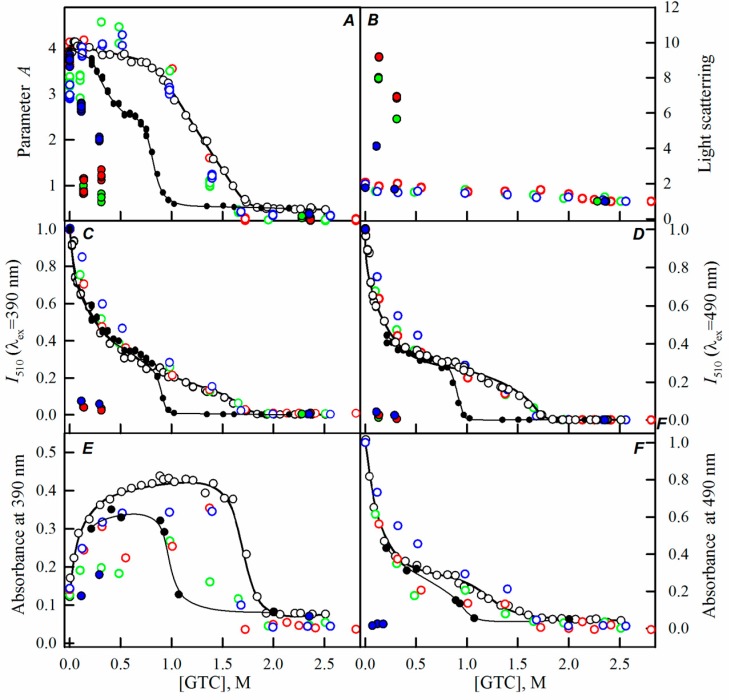
The effect of crowding agent Ficoll-70 on the conformational changes of sfGFP induced by GTC. For more details see the legend of Figure 2. Applied concentrations of Ficoll-70 were 8% (blue circles), 20% (green circles), and 30% (red circles).

Here, we have registered the dependencies of the different spectral characteristics of sfGFP during the GTC-induced unfolding-refolding processes in the presence of different crowding agents of different concentrations to test effect of crowding agents in a wide range of sizes on sfGFP stability and folding. The experimental data in the presence of PEGs of different molecular weights are presented in the Figure 2, Figure 3 and Figure 4, and for the Dextran-70 and Ficoll-70 are shown in the Figure 5 and Figure 6, respectively.

Denaturation dependencies of all registered characteristics of sfGFP in the presence of all tested concentrations of Dextran-70 (Figure 5) and Ficoll-70 (Figure 6) coincided with the unfolding curves of sfGFP in the absence of crowding agents.

However, PEGs of various molecular weights differently affected the unfolding-refolding processes of sfGFP. In the presence of PEG-600 (Figure 4) and PEG-8000 (Figure 3), the unfolding curves of sfGFP were shifted to a higher denaturant concentrations compared to sfGFP denaturation curves in buffered solution without crowding agents. Furthermore, this shift toward higher denaturant concentrations elevated with the increase in the concentration of crowding agent. Therefore, PEG-600 and PEG-8000 had concentration-dependent stabilizing effects on the structure of sfGFP. Contrary, PEG-12000 had both a destabilizing and a stabilizing effect on sfGFP. The addition of this crowding agent in a low concentration (8%, Figure 2) was followed by the shift of sfGFP denaturation curves to lower concentrations of the denaturant. At concentrations exceeding 20%, similarly to the PEG-600 and PEG-8000, PEG-12000 had a stabilizing effect on the structure of sfGFP and this effect was concentration-dependent.

**Figure 7:**
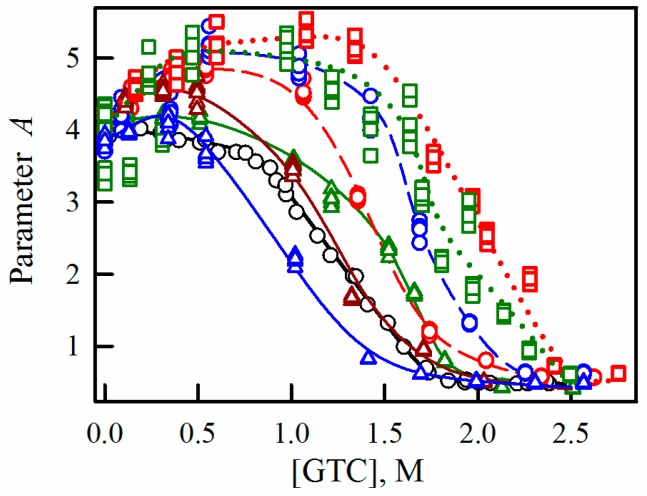
The effect of PEGs of different molecular weights on the denaturation of sfGFP induced by GTC. The change of parameter *A* (*A* = *I*_320_/*I*_365_) on GTC in the presence of PEG-600, PEG-8000, and PEG-12000 (designations are given in red, green and blue, respectively). The applied concentrations of PEGs were 8%, 20%, and 30% presented as triangles (and solid line), circles (and dashed line) and squares (and dotted line), respectively.

In order to compare the impact of PEGs with different molecular weight on the unfolding process of sfGFP, the denaturation curves of the protein in the presence of these agents were put together in the same plots. Figure 7 and Figure S5 show that at the concentration of 8%, PEG-600 stabilized the structure of sfGFP slightly, while the stabilizing effect of PEG-8000 was more pronounced, and PEG-12000 had a noticeable destabilizing effect on the sfGFP structure. When the concentration of crowding agents were raised to 30%, the impact exerted by PEGs on the protein changed. PEG-600 had the most pronounced stabilizing effect on the structure of sfGFP. Further, with increasing of the molecular weight of the crowding agent, the extent of its stabilizing effect on the protein was reduced. At the concentration of 20%, all PEGs stabilized sfGFP, with a more pronounced effect being induced by the crowding agents with higher molecular weights.

With the increase in the molecular weight and concentration of PEGs, an increase in the light scattering was observed, indicating the aggregation of the native sfGFP molecules on the pathway of the protein unfolding, whereas the addition of Dextran-70 or Ficoll-70 did not lead to the aggregation of native sfGFP molecules in the unfolding process of this protein (Figure 2, Figure 3, Figure 4, Figure 5 and Figure 6).

Reversibility of the GTC-induced unfolding of sfGFP was affected in the presence of all crowding agents used in this study. In fact, the protein failed to recover spectral characteristics recorded for the native protein prior to its unfolding (Figure 2, Figure 3, Figure 4, Figure 5 and Figure 6). This indicated the irreversibility of the sfGFP unfolding in the presence of crowding agents. It is worth noting that an increase in the light scattering was detected at sfGFP refolding in the presence of all crowding agents, indicating that the protein refolding process under these conditions is complicated by protein aggregation (Figure 2, Figure 3, Figure 4, Figure 5 and Figure 6). The strongest protein aggregation was observed in the presence of PEGs of all molecular weights (Figure 2, Figure 3 and Figure 4), whereas the aggregation of sfGFP during refolding was less pronounced in the presence of Dextran-70 (Figure 5 and Figure 6). Notably, the protein aggregation was stronger at the refolding of sfGFP compared to the unfolding pathway of this protein.

## 3. Discussion

The most common explanation of the effects of macromolecular crowding on various physiological processes is provided by the excluded volume theory. According to this theory, inside the limited space caused by macromolecular crowding, the equilibrium in the protein folding processes is shifted to more compact and more folded forms of a protein [24,27,28,29]. Thus, the macromolecular crowding favors the formation of the native form of a protein during its folding. It is believed that while modeling the crowding milieu with different crowding agents, their effects on the protein depend on the molecular mass of the crowder and its concentration and power with the increase of the polymer concentration [39]. The efficiency of macromolecular crowding is expected to be highest at conditions where hydrodynamic dimensions of a crowding agent are comparable to those of a tested protein [40,41]. In addition to the effect of excluded volume, the presence of a crowding agent in a solution may affect the properties of a tested protein in some other ways. In the presence of some direct interactions of a target protein with a crowding agent, the excluded volume effects can be either strengthened or weakened [42,43].

In this study, PEGs of different molecular weights, together with Ficoll-70 and Dextran-70, were applied to test the effects of variously-sized crowding agents on the stability and folding of sfGFP. Here, we tested the crowding agents with sizes ranging from about 6 Å to more than 60 Å. The Stokes radius of 23.8 Å of native sfGFP is in the middle of this range. The unfolded sfGFP is a more expanded protein form with a Stokes radius of 48.1 Å. The excluded volume theory suggests that the crowded environment should promote protein folding through an indirect stabilizing effect on the folded states of proteins by favoring more compact over less compact species. Within the frame of this excluded volume theory, this folding promoting effect of crowed media should increase with the increase in the crowding agent concentration and be more profound for the crowding agent with the hydrodynamic dimensions similar to those of a query protein. We observed more complex dependence of sfGFP stability and refolding on the presence of crowding agents. In fact, our previous data of the analysis of the urea-induced unfolding of structurally-different globular proteins [33,34], and the results of this study allow the conclusion that even in the absence of direct interaction between a crowding agent and the target protein the excluded volume theory cannot always be used to explain the experimental data. In fact, we show here that all crowding agents used in this study do not affect the structure of sfGFP. In the presence of high concentrations of PEG-12000, the observed changes of the spectral characteristics of sfGFP are caused by the protein aggregation rather than by a change of protein’s spatial structure. These data indicate that sfGFP does not interact directly with the crowding agents used here.

The presence of Ficoll-70 and Dextran-70 do not affect the conformational stability of sfGFP (it does not lead to the detectable shifts of the unfolding curves registered at the GTC-induced unfolding of this protein). Notably, the effective hydrodynamic radii of Ficoll-70 and Dextran-70 are 49.3 and 63.9 Å, respectively (calculated according to the formulas from [44]). The β-barrel of sfGFP is characterized by a diameter of 24 Å and a height of 42 Å [37], and native GFP (a globular protein with the molecular mass of 26,886 Da) is expected to have a Stokes radius of 23.8 Å, whereas the completely unfolded form of this protein is expected to have a Stokes radius of 48.1 Å [45]. Therefore, the lack of the evident influence of Ficoll-70 and Dextran-70 on the sfGFP unfolding-refolding processes and on the conformational stability of this protein is in apparent contradiction with the excluded volume theory, according to which the presence of high concentrations of crowding agents is expected to have an impact on the structural and conformational properties of a target protein.

The hydrodynamic radii of PEG-600, PEG-8000, and PEG-12000 are 5.6 Å ± 0.3 Å, 24.5 Å ± 1.9 Å, and 30.9 Å ± 2.5 Å, respectively [28]. According to the excluded volume theory, the greatest effect on the unfolding-refolding processes should be provided by PEG-8000, whose hydrodynamic dimensions are closest to those of sfGFP. According to our data, although PEGs influenced the conformational stability of sfGFP against the denaturing action of GTC, the impact of these crowding agents depended on the PEG molecular mass and concentration in a complex manner. At the PEG concentration of 8%, the crowder with the highest molecular weight caused some destabilization of the sfGFP structure, whereas the other two PEGs increased the resistance of the protein toward the GTC-induced unfolding. At concentrations of these crowding agents of 20%, their stabilizing effect on the sfGFP structure increased with the molecular weight of PEG. Finally, at the highest concentration of crowding agents (30%), PEG of lower molecular weight had the most pronounced stabilizing effect on sfGFP. The diversity of the effects of PEGs on the stability of sfGFP was not determined by the difference in interaction of this protein with the crowding agents, since none of the crowders used in our study caused noticeable changes in the spectral properties of sfGFP.

Our data indicated that during sfGFP refolding in the presence of all crowding agents used in this study, the recovery of protein characteristics to a level of the native protein was not observed even under strongly refolding conditions. The increase in the parameter *A* of the protein is insignificant, while the recovery in chromophore fluorescence was not detected. Furthermore, in the presence of all crowding agents, the changes of parameter *A* of sfGFP during refolding correlates well with the changes in the light scattering of the protein. In the intermediate state, the structure around the chromophore of sfGFP is fully recovered, while the environment of the tryptophan residue of sfGFP slightly differs from that in the native state of this protein [15]. These observations allowed us to conclude that during sfGFP refolding in the crowded milieu, the native or intermediate protein states were not formed. The observed changes of sfGFP characteristics can be attributed to the increase in the rigidity of the tryptophan environment caused by the aggregation of denatured protein molecules.

It is worth mentioning that in avGFP, the hydrogen bond network that involves Ser65 and Tyr66 residues of the chromophore, a water molecule, and the Ser205 and Glu222 residues from the chromophore’s close environment is known to play a crucial role in the chromophore maturation and the FP fluorescent properties’ acquisition [16,46,47]. Reversely, the chromophore strongly contributes not only to the protein refolding processes, but also to the protein conformational stability, by holding a compact structure of the β-barrel through the non-covalent interactions with the surrounding protein matrix [15]. Based on the Fourier transform infrared study on the state of water sorbed to PEG films it has been concluded that this polymer (which was used as one of the crowding agents in our study) can not only sorb the water molecules (where water molecules bind to the oxygen atoms of PEG with one of their hydrogen atoms leading to the formation of “binding water”), but can also affect the dynamic behavior of water by forming a dimeric water molecule by the association of the free water molecule with the binding water [48]. Therefore, it is likely that the sorption of the water molecules by the crowding agent may result in the destruction of the hydrogen network in sfGFP, affecting conformational stability and refolding of this protein.

## 4. Materials and Methods

### 4.1. Materials

GTC (Fluka, Buchs, Switzerland) and crowding agents PEG-600, PEG-8000, and PEG-12000, Dextran-70, and Ficoll-70 (Sigma-Aldrich, St. Louis, MO, USA) were used without additional purification. The absorbance of protein samples did not exceed 0.15 in all our experiments unless otherwise indicated. Taking into account the extinction coefficient ɛ_280_ = 31,519 M^−1^·sm^−1^ [19], this means that protein concentration was not higher than 5 µM. Measurements were conducted in 100 mM Na-phosphate-buffered solution at pH 8.0 in the absence or presence of different concentrations of crowding agents.

### 4.2. Gene Expression and Protein Purification

Construction of the plasmid (pET-28a(+)-sfGFP) coding for the poly-histidine tag containing super-folder GFP (sfGFP, [10]) was conducted following the previously described protocols [49]. *Escherichia coli* BL21 (DE3) cells (Invitrogen, Waltham, MA, USA) were used for transformation, and the sfGFP expression was induced by adding 0.5 mM isopropyl-β-d-1-thiogalactopyranoside (IPTG; Fluka, Buchs, Switzerland) with subsequent incubation for 24 h at 23 °C. His-GraviTrap columns (GE Healthcare, Little Chalfont, UK) packed with Ni^+^-agarose were used for the purification of the recombinant protein. The 15% polyacrylamide gel electrophoresis in the presence of sodium dodecyl sulfate SDS-PAGE [50] revealed that purity of the sfGFP was at least 95%.

### 4.3. Spectrophotometric Experiments

An EPS-3T spectrophotometer (Hitachi, Tokyo, Japan) with microcells 101.016-QS 5 × 5 mm (Hellma, Mulheim, Germany) was used for the absorption measurements, all of which were performed at room temperature.

### 4.4. Fluorescence Spectroscopy

A Cary Eclipse spectrofluorometer with microcells (10 × 10 mm; Agilent Technologies, Mulgrave, Victoria, Australia) equipped with a thermostat was used for conducting fluorescence experiments at the constant temperature of 23 °C. A recently proposed approach was used for the correction of the fluorescence intensity for the primary inner filter effect [51,52].

To minimize the contribution of tyrosine residues in the bulk protein fluorescence, the long-wave absorption spectrum edge (λ_ex_ = 295 nm) was used for excitation of the protein tryptophan fluorescence. The effects of environmental factors on the position and shape of the fluorescence spectra were analyzed using the *A* = *I*_320_/*I*_365_ parameter, with *I*_320_ and *I*_365_ being the fluorescence emission intensities at the wavelengths of 320 and 365 nm, respectively. The fluorescence intensity and the parameter *A* values were corrected for the instrument sensitivity. The light scattering was recorded at the coincident excitation and registration wavelengths (of 295 nm). To analyze the fluorescent properties of the neutral and anionic forms of the “green” chromophore, the corresponding fluorescence these two forms was exited at 390 and 490 nm and recorded at 450 and 510 nm, respectively.

The unfolding of the protein was initiated by the manual mixing of a native protein solution (50 µL) with a buffer solution (450 µL). In addition to the desired concentrations of guanidine thiocyanate (GTC) the buffer in these experiments contained desired concentrations of a crowding agent. An Abbe refractometer (LOMO, Saint-Petersburg, Russia) was used to measure the refraction coefficient values needed for the determination of the GTC concentrations in stock solutions. To initiate sfGFP refolding, the 50 µL aliquot of the pre-unfolded (in 2.5 M GTC) protein was mixed with 450 µL of buffer or solutions containing desired concentrations of the denaturant and a crowding agent. Protein solutions containing a desired denaturant concentration, with or without crowding agent at the desired concentration, were incubated at 23 °C for 24 h. It is worth noting that thus-measured characteristics of the protein are quasi-equilibrium parameters [15]. According to the control experiments, the dead-time in these manual mixing-based unfolding-refolding experiments was about 4 s [53,54].

### 4.5. Circular Dichroism Measurements

A thermostat-equipped Jasco-810 spectropolarimeter (Jasco, Tokyo, Japan) was used for recording the protein circular dichroism (CD) spectra with a step size of 0.1 nm. Far-UV (in the range of 250–190 nm), near-UV (in the 320–250 nm range), and visible CD spectra (in the range from 550 to 320 nm) were recorded using the 1-mm (far-UV CD) and 10-mm path length cells (near-UV and visible CD), respectively. An average of three scans was obtained for of all of the protein CD spectra, which were corrected by subtracting the CD spectra of the appropriate buffer solution.

## 5. Conclusions

Data reported in this study suggest that, in addition to the expected excluded volume effects, crowding agents can also cause some changes in the properties of the solvent. This hypothesis is supported by the observed increase in the degree of sfGFP aggregation during the protein unfolding-refolding processes with the increase in the molecular mass and concentration of PEGs. In fact, it is evident now that the behavior of biomolecules in the conditions of macromolecular crowding cannot be explained only within the frames of the excluded volume effects and/or direct interaction between tested biomolecules and crowding agents [28,29,33,34]. The changes in the sfGFP stability towards the GTC action and the capability of this protein to refold in the presence of different crowding agents are shown here to be dependent on the type and concentration of the polymer used as a crowding agent. Furthermore, in the case of PEG, the effects of the crowder is further influenced by the molecular mass of the polymer. This further supports the idea that, in addition to the excluded volume, some additional factors are involved in the modulation of the behavior of sfGFP in the crowded milieu. Accumulated experimental data including results obtained in this study indicate that crowding agents may affect the solvent properties of aqueous solutions in different manners, depending on the crowding agent macromolecule structure and the concentration [33,34,55]. These solvent properties are the solvent dipolarity/polarizability, hydrogen-bond donor acidity, and hydrogen-bond acceptor basicity of aqueous solutions [55]. The diverse impact of different polymers on the solvent properties of water leads to the unequal changes of the protein properties in the crowded environment modeled by these polymers. Therefore, restructuring of aqueous media might contribute greatly to the biological processes in a crowded milieu.

## Supplementary Materials

Supplementary materials can be found at www.mdpi.com/1422-0067/17/11/1805/s1.
