# Supplementary material for: Peculiarities of the Super-Folder GFP Folding in a Crowded Milieu
Source: Int J Mol Sci. 2016 Oct 28;17(11):1805. doi: 10.3390/ijms17111805 (PMC5133806; doi:10.3390/ijms17111805)
Supplement: Supplementary file 1 [file ijms-17-01805-s001.pdf]

Supplementary Materials: Peculiarities of the Super-Folder GFP Folding in a Crowded Milieu

Olesya V. Stepanenko, Olga V. Stepanenko, Irina M. Kuznetsova, Vladimir N. Uversky and Konstantin K. Turoverov

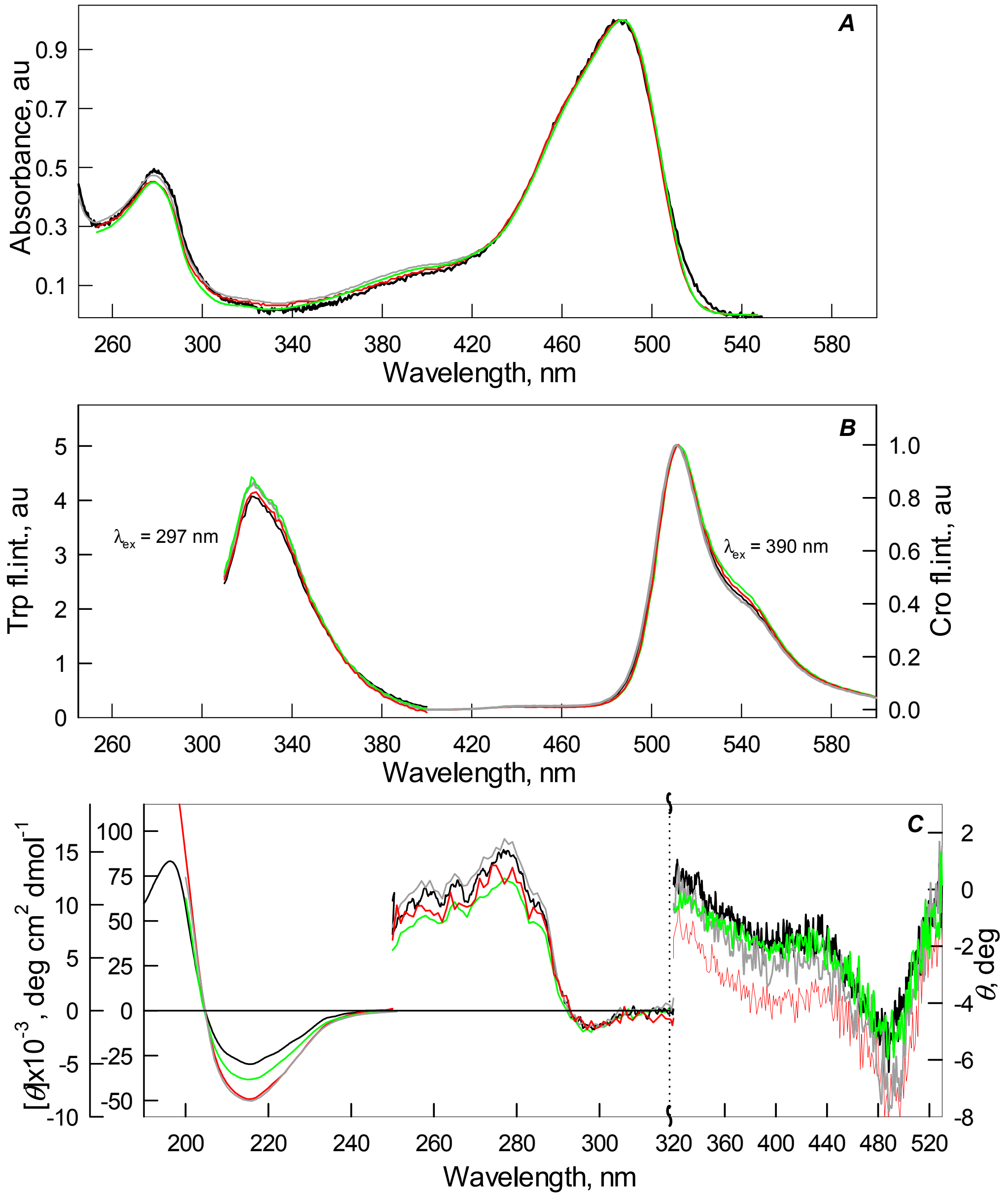

Figure S1. The effect of crowding agent Dextran-70 on the spectral properties of sfGFP. Absorption spectra (**A**); tryptophan fluorescence spectra at λ_{ex} = 295 nm and green fluorescence spectra at λ_{ex} = 390 nm (**B**); and CD spectra in the far (the data are represented in the units of the molar ellipticity per amino acid residue), near (in the units of the molar ellipticity) and visible UV-region (in the units of the ellipticity) were recorded (**C**). Applied concentrations of Dextran-70 were 8% (green line), 24% (gray line) and 30% (red line). The spectra of sfGFP in the buffered solution (black lines) are shown for comparison purposes.

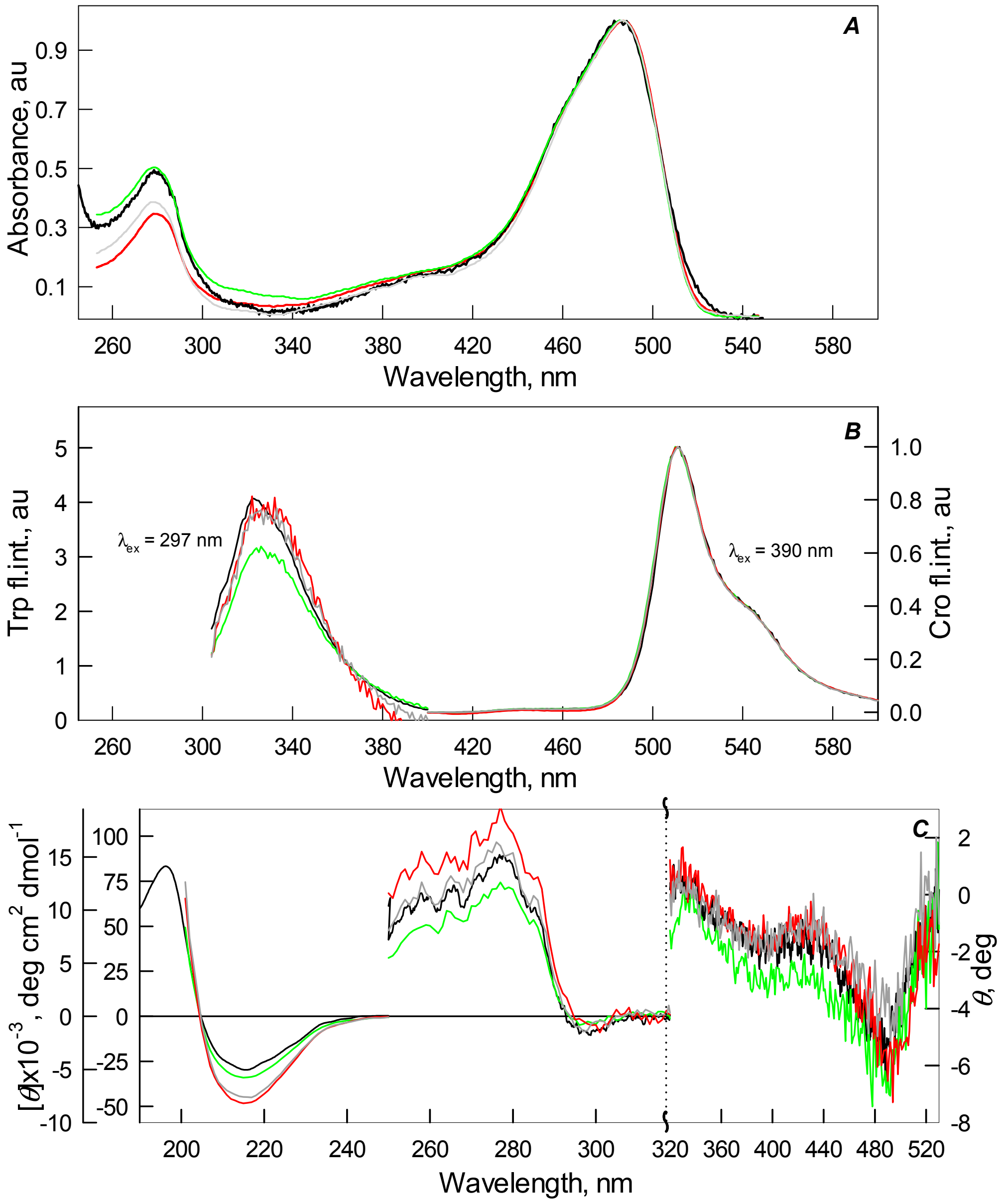

Figure S2. The effect of crowding agent Ficoll-70 on the spectral properties of sfGFP. For more details see the legend of Figure S1. Applied concentrations of Ficoll-70 were 8% (green line), 20% (gray line) and 30% (red line).

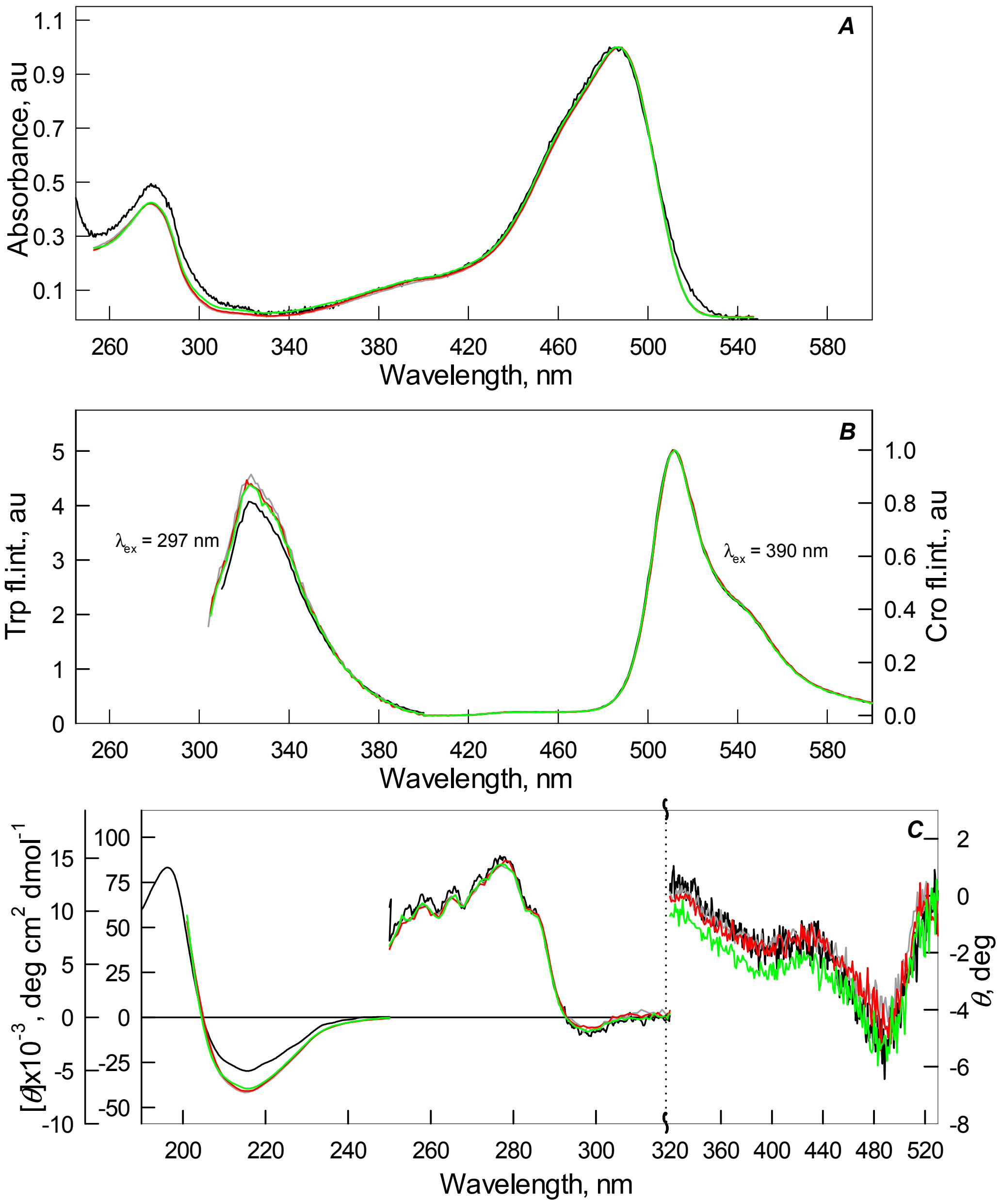

Figure S3. The effect of crowding agent PEG-600 on the spectral properties of sfGFP. For more details see the legend of Figure S1. Applied concentrations of PEG-600 were 8% (green line), 20% (gray line) and 30% (red line).

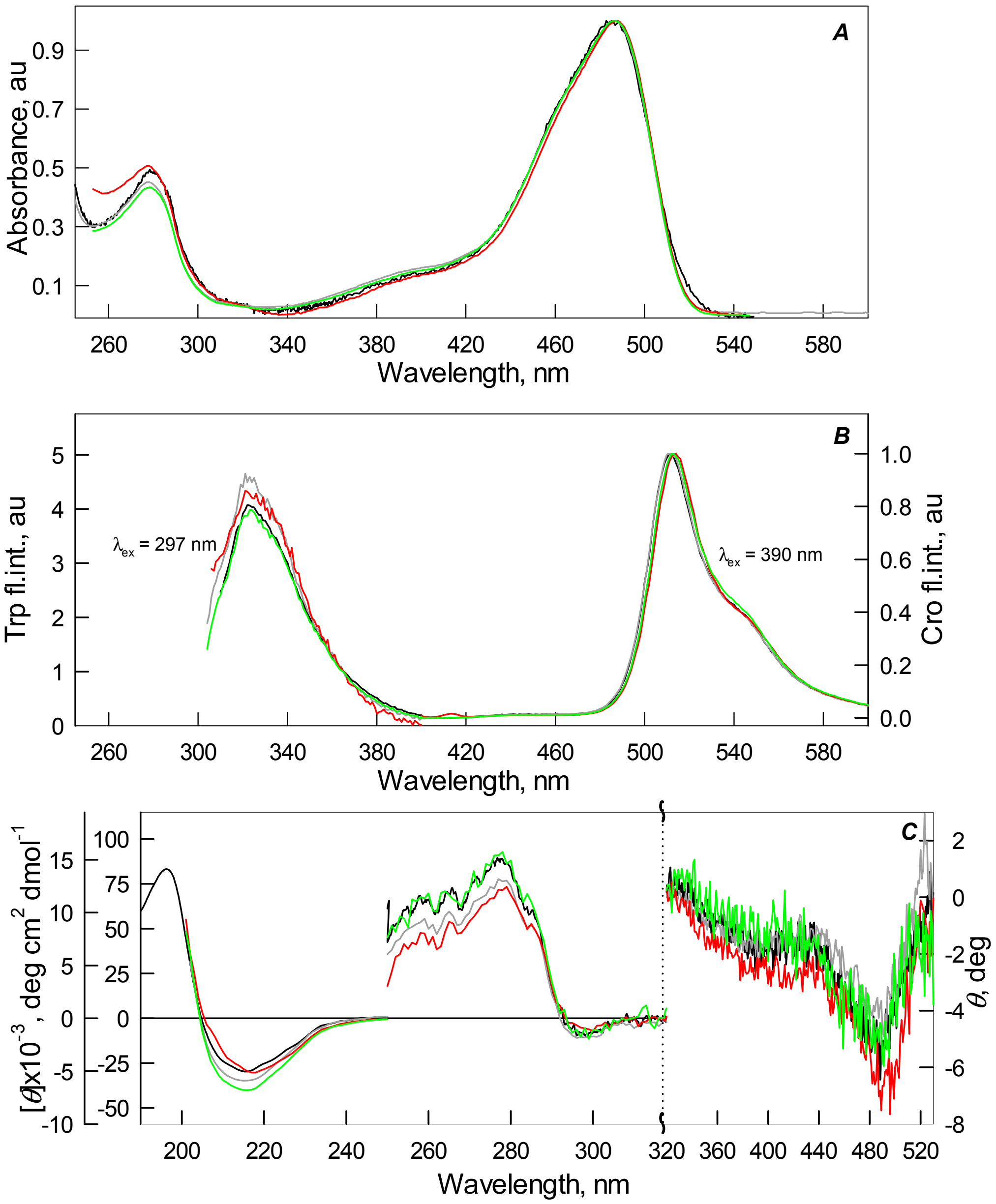

Figure S4. The effect of crowding agent PEG-8000 on the spectral properties of sfGFP. For more details see the legend of Figure S1. Applied concentrations of PEG-8000 were 8% (green line), 12% (gray line) and 30% (red line).

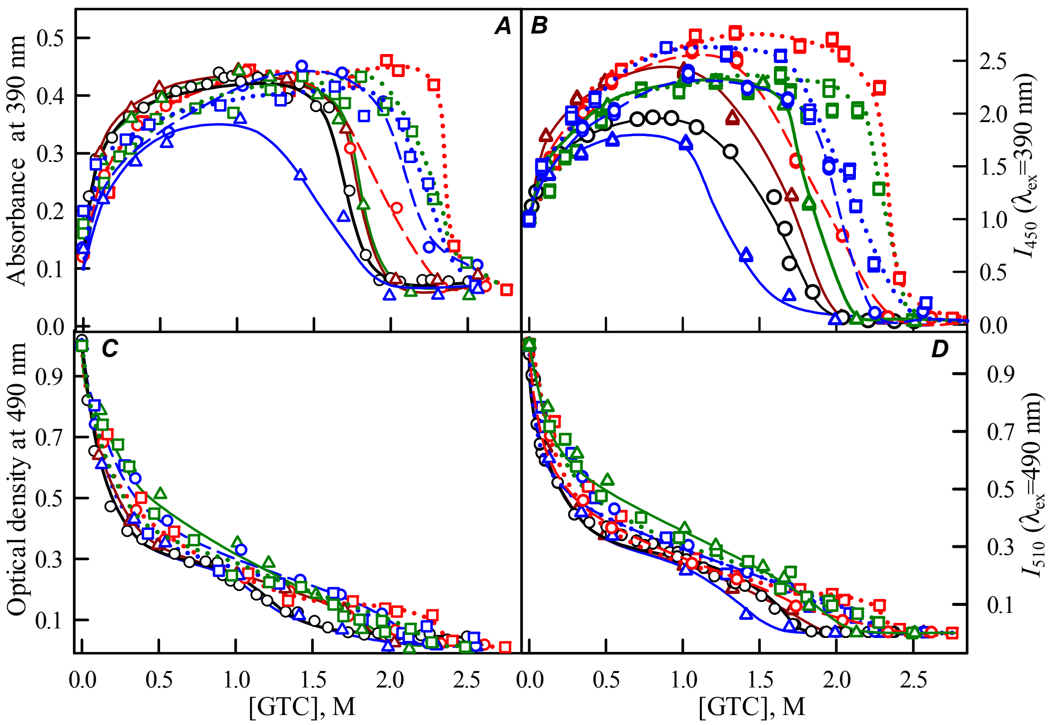

Figure S5. The effect of PEGs of different molecular weights on the denaturation of sfGFP induced by GTC. Changes of absorbance at 390 nm (**A**) and 490 nm (**C**); changes in direct fluorescence from neutral form of the green chromophore at 450 nm at excitation wavelength of 390 nm (**B**); and changes in fluorescence intensity from anionic form of the green chromophore at excitation at 490 nm (**D**) are shown. Measurements were performed after 24 h incubation of native protein in the presence of GTC (black circles and solid line) or GTC and crowding agents PEG-600, PEG-8000 and PEG-12000 (designations are given in red, green and blue, respectively). The applied concentrations of PEGs were 8%, 20% and 30% presented as triangles (and solid line), circles (and dashed line) and squares (and dotted line), respectively.
